# Age-Dependent Targeting of Protein Phosphatase 1 to Ca^2+^/Calmodulin-Dependent Protein Kinase II by Spinophilin in Mouse Striatum

**DOI:** 10.1371/journal.pone.0031554

**Published:** 2012-02-13

**Authors:** Anthony J. Baucum, Stefan Strack, Roger J. Colbran

**Affiliations:** Department of Molecular Physiology and Biophysics, Vanderbilt-Kennedy Center, Center for Molecular Neuroscience, Vanderbilt University School of Medicine, Nashville, Tennessee, United States of America; Roswell Park Cancer Institute, United States of America

## Abstract

Mechanisms underlying age-dependent changes of dendritic spines on striatal medium spiny neurons are poorly understood. Spinophilin is an F-actin- and protein phosphatase 1 (PP1)-binding protein that targets PP1 to multiple downstream effectors to modulate dendritic spine morphology and function. We found that calcium/calmodulin-dependent protein kinase II (CaMKII) directly and indirectly associates with N- and C-terminal domains of spinophilin, but F-actin can displace CaMKII from the N-terminal domain. Spinophilin co-localizes PP1 with CaMKII on the F-actin cytoskeleton in heterologous cells, and spinophilin co-localizes with synaptic CaMKII in neuronal cultures. Thr286 autophosphorylation enhances the binding of CaMKII to spinophilin *in vitro* and *in vivo*. Although there is no change in total levels of Thr286 autophosphorylation, maturation from postnatal day 21 into adulthood robustly enhances the levels of CaMKII that co-immunoprecipitate with spinophilin from mouse striatal extracts. Moreover, N- and C-terminal domain fragments of spinophilin bind more CaMKII from adult vs. postnatal day 21 striatal lysates. Total levels of other proteins that interact with C-terminal domains of spinophilin decrease during maturation, perhaps reducing competition for CaMKII binding to the C-terminal domain. In contrast, total levels of α-internexin and binding of α-internexin to the spinophilin N-terminal domain increases with maturation, perhaps bridging an indirect interaction with CaMKII. Moreover, there is an increase in the levels of myosin Va, α-internexin, spinophilin, and PP1 in striatal CaMKII immune complexes isolated from adult and aged mice compared to those from postnatal day 21. These changes in spinophilin/CaMKII interactomes may contribute to changes in striatal dendritic spine density, morphology, and function during normal postnatal maturation and aging.

## Introduction

Dendritic spines are small, actin-rich protrusions from neuronal dendrites that are sites of excitatory synaptic inputs. Spine density and morphology are regulated by synaptic activity [1], and are continuously modified during development and aging. For instance, dendritic spine density in feline caudate medium spiny neurons increases during maturation from postnatal days (PND) 1–50 into adulthood (1–3 years old) and then decreases during advanced age (13–18 years old) [2], [3]. There is a growing understanding of molecules and pathways that contribute to changes in spine density and morphology during development (for review see [4]). Emerging *in vivo* imaging techniques are also revealing dynamic changes in dendritic spines in response to synaptic activity and during aging [5]. However, there is a relatively poor understanding of the proteins and *in vivo* mechanisms that control spine changes in the developing and mature brain.

The postsynaptic density (PSD) is localized at the tips of dendritic spine heads and contains multiple classes of proteins that mediate neuronal signal transduction in response to presynaptic glutamate release [6]. Calcium/calmodulin-dependent protein kinase II (CaMKII), a serine/threonine kinase, is one of the most abundant proteins localized to forebrain PSDs [7]. CaMKII regulates synaptic strength, in part by phosphorylating glutamate receptors [8], [9]. CaMKII also has protein scaffolding functions in dendritic spines [10]. Autophosphorylation of CaMKIIα at Thr286 leads to autonomous kinase activity, stabilizes CaMKII localization at the PSD, and is essential for normal learning and memory [11], [12], [13], [14]. CaMKIIα levels increase during normal postnatal development and are also increased in a genetic mouse model of accelerated aging [15], [16]. Moreover, in aged animals, oxidative stress can regulate glutamate receptor activity in a CaMKII-dependent manner [17]. Given the critical role of CaMKII as a signaling and scaffolding molecule, it seems likely that CaMKII will play a key role in controlling spine morphology, density, and function during aging.

One key regulator of CaMKII is protein phosphatase 1 (PP1) [13], a serine-threonine phosphatase that is also localized to dendritic spines and PSDs [18], [19]. Inhibition of PP1 enhances Thr286 autophosphorylation of CaMKIIα during the induction of long-term potentiation [20] and also increases synaptic strength in hippocampus of aged, but not young adult, animals [21]. Moreover, striatal dopamine depletion decreases PP1 activity and increases CaMKIIα autophosphorylation at Thr286 [22], [23]. Therefore, changes in the activity and subcellular targeting of PP1 can modulate CaMKII and other PSD proteins.

The physiological functions of PP1 and CaMKII are modulated by a variety of binding proteins (for reviews see [24], [25]). For example, spinophilin, and its homolog neurabin, are F-actin binding proteins that target PP1 to the PSD [26] and also bind several additional proteins that can modulate spine dynamics in neurons [27], [28], [29], [30]. Our recent, comprehensive proteomics analysis demonstrated that CaMKII is a component of adult striatal spinophilin complexes [31]. Here we report that spinophilin can target CaMKII to F-actin as well as target PP1 to CaMKII. This targeting is achieved by complex direct and indirect interactions of CaMKII with spinophilin. Interestingly, striatal CaMKII-spinophilin interactions increase during maturation and aging, enhancing the targeting of PP1 to CaMKII. These data provide new insight into the age-dependent modulation of dendritic protein complexes.

## Results

### Autophosphorylated CaMKII directly binds spinophilin at two different sites

**Figure 1:**
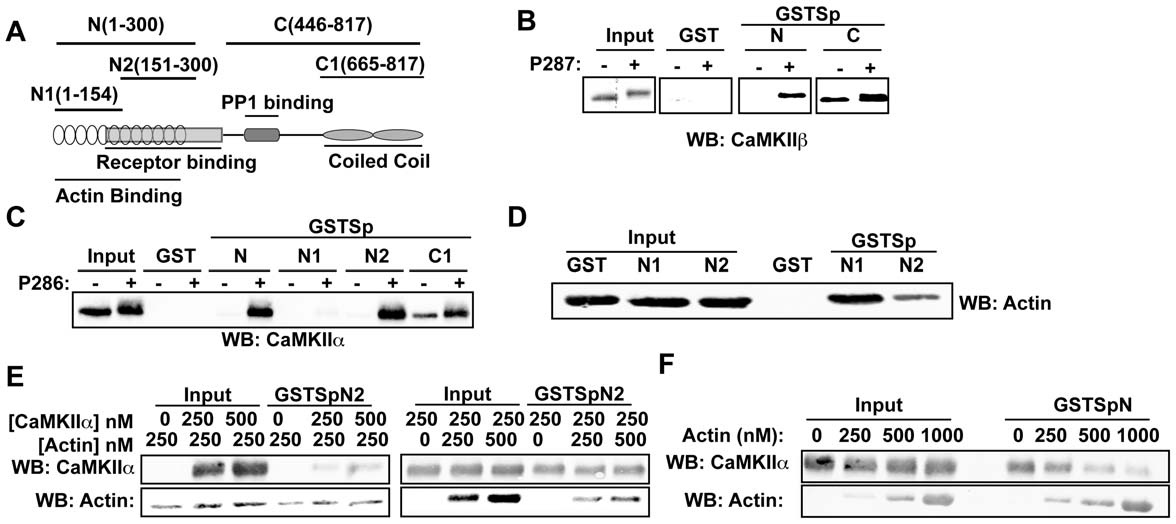
Multiple domains in spinophilin directly bind purified CaMKII isoforms. *A.* Stylized domain structure of spinophilin showing the F-actin-, G-protein coupled receptor-, and PP1-binding domains, as well as the coiled-coil domains. GST spinophilin fusion proteins containing the indicated fragments were generated. *B.* Non- or Thr287-autophosphorylated CaMKIIβ was incubated with GST, GSTSpN, or GSTSpC. Complexes were isolated using glutathione agarose and then immunoblotted for CaMKIIβ. *C.* Non- or Thr286-autophosphorylated CaMKIIα was incubated with GST or the indicated GST-fusion proteins. Complexes were isolated as in Panel B and immunoblotted for CaMKIIα. *D.* F-actin was incubated with GST, GSTSpN1, or GSTSpN2. Complexes were isolated using glutathione agarose and immunoblotted for actin. *E.* GSTSpN2 was incubated with CaMKII or F-actin in the presence of increasing concentrations of F-actin or CaMKII, respectively. Complexes were isolated using glutathione agarose and immunoblotted for CaMKII and actin. *F.* GSTSpN was incubated with CaMKIIα (250 nM) in the presence of increasing concentrations of F-actin. Complexes were isolated using glutathione agarose and immunoblotted for CaMKIIα and actin. All figures are representative of at least 2 experiments.

In order to test for a direct interaction of CaMKII with spinophilin, we incubated purified CaMKII with a family of GST-spinophilin fusion proteins containing different domains of spinophilin (Fig. 1A). Proteins containing residues 1–300 (GSTSpN) or residues 446–817 (GSTSpC) directly bound CaMKIIβ (Fig. 1B). CaMKIIβ autophosphorylation at Thr287 was required for binding to GSTSpN, whereas binding to GSTSpC was partially independent of autophosphorylation. Similarly, GSTSpN2 (residues 151–300), but not GSTSpN1 (residues 1–154), directly interacted with CaMKIIα in a Thr286 autophosphorylation-dependent manner (Fig. 1C), whereas binding of CaMKIIα to GSTSpC1 (residues 665–817) was partially independent of autophosphorylation. Comparison of data from multiple experiments suggested that autophosphorylated CaMKII isoforms displayed somewhat different selectivity between GSTSpN and GSTSpC. Purified CaMKIIβ consistently bound more robustly to GSTSpC than to GSTSpN, whereas purified CaMKIIα bound more robustly to GSTSpN than to GSTSpC.

The N-terminal domains of spinophilin have also been shown to bind to and bundle F-actin filaments [32], [33]. Therefore, we investigated whether F-actin affected the binding of CaMKII to N-terminal domains of spinophilin. GSTSpN1 robustly bound F-actin, whereas much lower amounts of F-actin bound to GSTSpN2 (Fig. 1D), consistent with a previous study [33]. Notably, F-actin failed to displace CaMKII from GSTSpN2 (Fig. 1E). However, F-actin displaced CaMKII from the larger N-terminal GSTSpN fragment in a concentration-dependent manner (Fig. 1F).

### Reduced binding of CaMKII to spinophilin in T286A-KI mice

**Figure 2:**
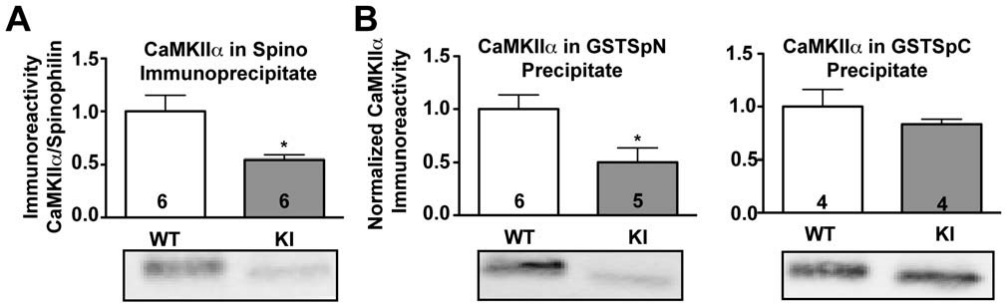
Spinophilin binding to CaMKIIα is decreased in T286A-KI mice. *A.* Spinophilin was immunoprecipitated from striatal TSFs of WT or T286A-KI mouse striatum and immune complexes were immunoblotted for CaMKIIα. *B.* Striatal TSFs from WT or T286A-KI mice were incubated with GSTSpN or GSTSpC. Complexes were isolated using glutathione agarose and immunoblotted for CaMKIIα.

In order to determine whether CaMKIIα autophosphorylation at Thr286 can regulate binding to spinophilin *in vivo*, we analyzed striatal extracts from mice with a knock-in mutation of Thr286 to Ala in CaMKIIα (T286A-KI). The T286A-KI mutation reduced levels of CaMKIIα in spinophilin immune complexes by ≈50% (Fig. 2A). Consistent with *in vitro* data (Fig. 1C), GSTSpN bound reduced levels of CaMKII from striatal extracts of T286A-KI compared to WT mice, whereas GSTSpC bound similar levels of CaMKII from extracts of WT and T286A-KI mice (Fig. 2B).

### Coiled-coil structure is necessary for CaMKII binding to C-terminal domains of spinophilin

**Figure 3:**
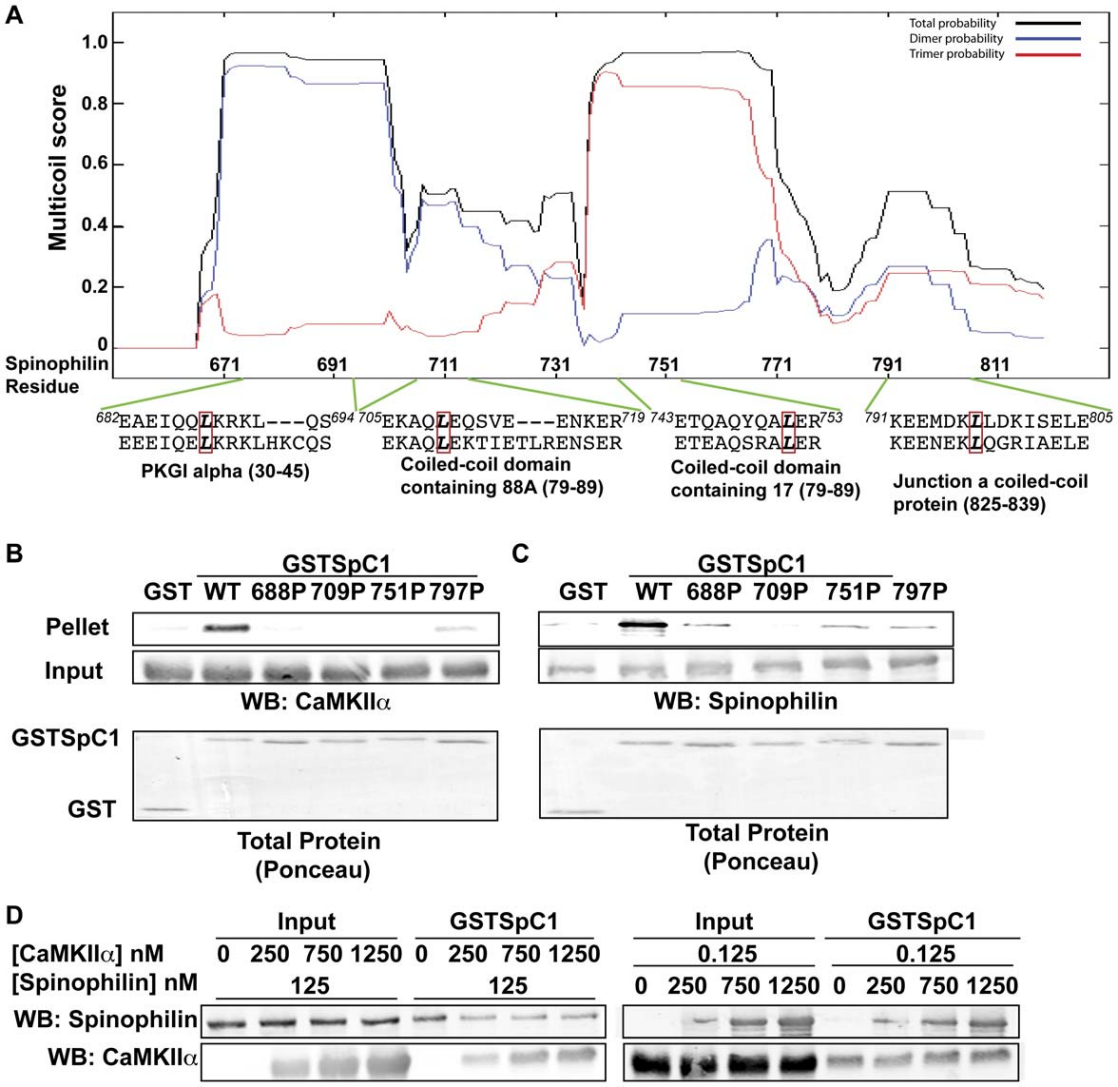
Role of C-terminal coiled-coil domains of spinophilin. *A.* The MultiCoil algorithm was used to predict coiled-coil domain structures in the C-terminal domain of spinophilin. Amino acid sequences of the four predicted coiled-coil domains in spinophilin were aligned with amino acid sequences of coiled-coil domains in other proteins as indicated below. Residues 682–694 with mouse PKGI alpha; residues 705–719 with mouse coiled-coil domain containing 88A; residues 741–751 with mouse coiled-coil domain containing 17; residues 791–805 with mouse junction a coiled coil protein. A red box indicates conserved leucines that were mutated to proline. These mutations were predicted to selectively disrupt each of the coiled-coil domains using Multicoil (Fig. S1). *B.* Non- or Thr286-autophosphorylated CaMKIIα was incubated with WT or mutated (L688P, L709P, L751P, or L797P) GSTSpC1. Complexes were isolated using glutathione agarose and GST precipitates were immunoblotted for CaMKII. The total protein stain (Ponceau) of GST or GST proteins is also shown. *C.* His-tagged full-length, WT spinophilin was incubated with WT or mutated (L688P, L709P, L751P, or L797P) GSTSpC1. GST precipitates were isolated using glutathione agarose and immunoblotted for spinophilin. Total protein stain (Ponceau) of GST or GST proteins is also shown *D.* Non phosphorylated CaMKII or spinophilin was incubated GSTSpC1 along with increasing concentrations of either spinophilin or CaMKII. Complexes were isolated using glutathione agarose and immunoblotted for spinophilin and CaMKII. Figures are representative of at least 2 experiments.

The spinophilin C-terminus was predicted to contain multiple coiled-coil domains that mediate its assembly into multimeric structures [34]. Mutation of leucine to proline within coiled-coil structures has been shown to disrupt protein-protein interactions [35]. The sequences of four predicted coiled-coil domains in the C-terminal region of spinophilin were aligned with sequences of coiled-coil domains from other proteins in order to identify conserved leucines that may be structurally important (Fig. 3A). The MultiCoil algorithm predicted that leucine to proline mutations (L688P, L709P, L751P, L797P) individually disrupt the predicted coiled-coil structures of each region [36] (Fig. S1A–D). Notably, each mutation reduced binding of either purified CaMKII or purified, full-length, WT, His-tagged spinophilin to GSTSpC1 (Fig. 3B, 3C). These data suggest that each coiled-coil structure in the C-terminal domain is required for both multimerization of spinophilin and for direct binding of purified CaMKII.

We next investigated whether oligomerization of spinophilin affects the interaction with CaMKII. Up to a 10-fold molar excess of CaMKII had no reliable effect on the binding of His-spinophilin to GSTSpC1 (Fig. 3D). Similarly, the binding of CaMKII to GSTSpC1 was not substantially affected by up to a 10-fold molar excess of full-length, WT, His-spinophilin (Fig. 3D). In combination, these data indicate that even though CaMKII binding to the spinophilin C-terminal domain requires an intact coiled-coil structure, the binding of CaMKII is largely unaffected by oligomerization of spinophilin.

### Spinophilin targets PP1 to CaMKII

**Figure 4:**
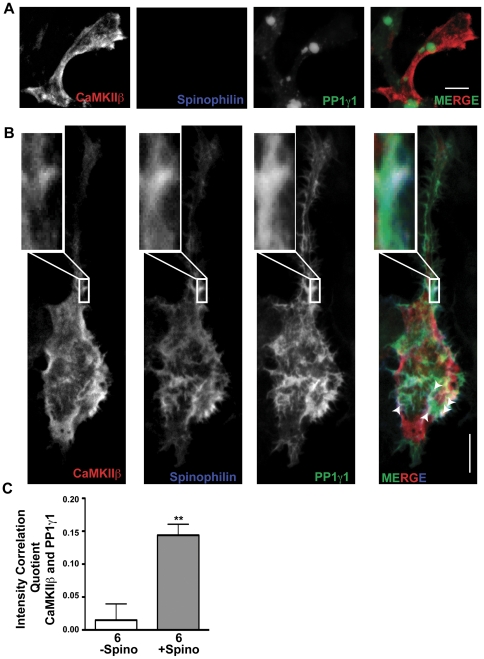
Spinophilin targets PP1γ1 to CaMKIIβ. *A.* Compressed Z-stack of confocal images of HEK293 cells expressing CaMKIIβ and GFP-tagged PP1γ1 in the absence of spinophilin. Scale bar: 10 mm *B.* Compressed Z-stack of confocal images of HEK293 cells expressing CaMKIIβ and GFP-PP1γ1 in the presence of spinophilin. White box shows zoomed in view of a cortical region containing all 3 proteins. Scale bar: 10 mM. *C.* Intensity correlation quotient showing significant co-localization of CaMKIIβ with GFP-PP1γ1 in the presence (+Spino), but not absence (−Spino), of spinophilin. Values are expressed as the mean±S.E.M from analyses of the indicated numbers of cells. ICQ values were derived from cells imaged from 2 separate sets of transfections. **P<0.01.

In order to determine whether spinophilin can play a role in targeting PP1 to CaMKII, we co-expressed GFP-PP1γ1 and untagged WT CaMKIIβ in HEK293 cells with or without spinophilin. GFP-PP1γ1 adopted a predominantly perinuclear localization in the absence of co-expressed spinophilin, as seen previously [37], whereas CaMKIIβ was concentrated at the cortical sub-membrane cytoskeleton, presumably due to the presence of a previously defined F-actin-binding domain [38], [39], [40] (Fig. 4A). However, co-expression of spinophilin induced a substantial redistribution of PP1γ1 to cortical regions of the cell, as seen previously [37], such that PP1γ1 now co-localized with spinophilin and CaMKIIβ in some of the cortical regions (white arrows in Fig. 4B and additional HEK cell images in Fig. S2 and S3). Semi-quantitative analysis of these images using an intensity correlation approach (see Methods) revealed significant co-localization of PP1γ1 with CaMKIIβ in the presence, but not the absence, of spinophilin (Fig. 4C). These data suggest that spinophilin can target PP1 to CaMKII.

### Spinophilin targets CaMKII to F-actin in heterologous cells

**Figure 5:**
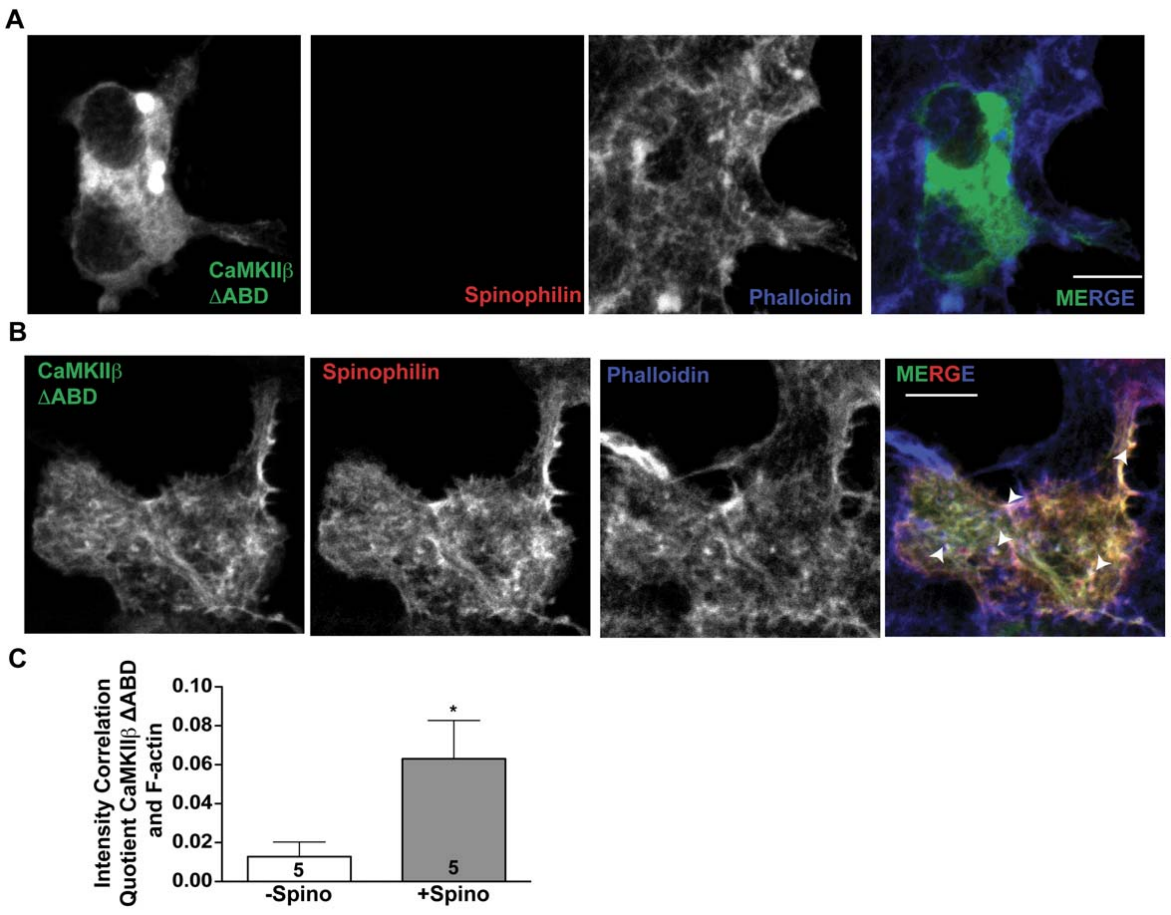
Spinophilin targets CaMKII to F-actin. A. Compressed Z-stack of confocal images of HEK293 cells expressing GFP-tagged CaMKIIβ ΔABD. F-actin was detected using a far-red phalloidin stain. Scale bar: 10 mM. B. Compressed Z-stack of confocal images of HEK293 cells expressing GFP-tagged CaMKIIβ ΔABD and myc-spinophilin. F-actin was detected using a far-red phalloidin stain. Scale bar: 10 mM. C. Intensity correlation quotient showing significant co-localization of CaMKIIβ with phalloidin in the presence (+Spino), but not absence (−Spino), of spinophilin. Values are expressed as the mean±S.E.M from analyses of the indicated numbers of cells. * P<0.05.

In order to determine whether spinophilin can target CaMKII to the F-actin cytoskeleton, we expressed GFP-tagged CaMKIIβ lacking the intrinsic F-actin binding domain (ΔABD) in HEK293 cells with or without co-expressed spinophilin. In the absence of spinophilin, GFP-CaMKIIβ ΔABD was rather diffusely localized in the cytoplasmic regions of the cell, overlapping poorly with phalloidin-stained F-actin (Fig. 5A). Co-expression of spinophilin resulted in a pronounced cortical localization of GFP-CaMKIIβ ΔABD that overlapped with both spinophilin and phalloidin-stained structures (Fig. 5B). Intensity correlation analysis revealed significant co-localization between spinophilin and GFP-CaMKIIβ ΔABD (ICQ value = 0.20±0.035; N = 5), and significantly more co-localization between CaMKIIβ ΔABD and F-actin in the presence, compared to the absence, of spinophilin (Fig. 5C). In combination, these data suggest that spinophilin can target CaMKII to the F-actin cytoskeleton independent of direct CaMKII binding to F-actin.

### Spinophilin co-localizes with CaMKII in neurons

**Figure 6:**
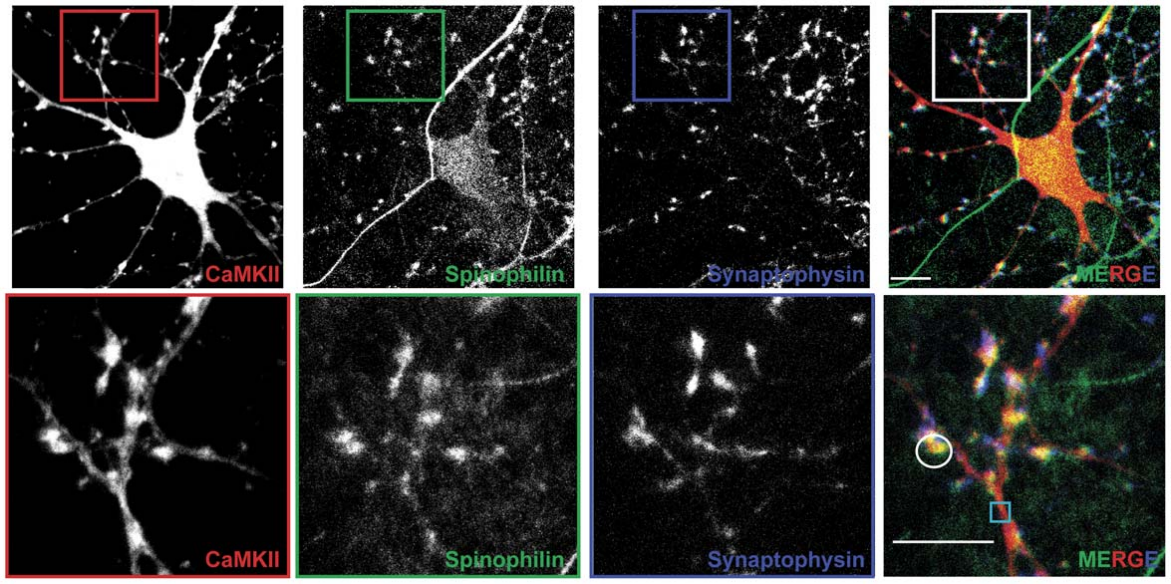
Spinophilin co-localizes with CaMKII in neurons. Confocal images of CaMKII (red), spinophilin (green), and synaptophysin (blue) immunofluorescence. Boxes below show higher magnification of a dendritic section. Scale bars:10 mM.

We previously showed that PP1γ1 co-localizes with CaMKII in dendritic spines of cultured neurons [19]. In order to determine whether spinophilin co-localizes with CaMKII in dendritic spines, we immunostained cultured neurons for spinophilin and CaMKII. Spinophilin and CaMKII co-localized predominantly in dendritic spines, overlapping with or adjacent to presynaptic terminals containing synaptophysin (white circle in Fig. 6), with little co-localization in dendritic shafts (cyan box in Fig. 6). These data demonstrate that spinophilin and CaMKII co-localize at F-actin-rich postsynaptic sites in neurons.

### Maturation increases the association of CaMKII with spinophilin

**Figure 7:**
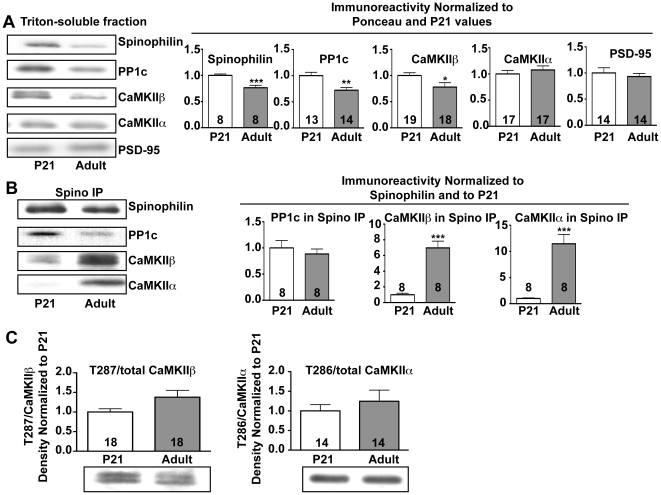
The interaction of CaMKII with spinophilin increases with age. *A.* TSFs isolated from PND21 or adult striatum were immunoblotted for spinophilin, PP1 catalytic subunit (PP1c), CaMKIIβ, CaMKIIα, or PSD-95. *B.* Spinophilin was immunoprecipitated from TSFs isolated from PND21 (P21) or adult animals. Precipitates were immunoblotted for spinophilin, PP1c, CaMKIIβ, or CaMKIIα. *C.* Striatal TSFs from PND21 or adult mice were immunoblotted for phospho-Thr286/7 and total CaMKII. Phospho-Thr286/7 immunoreactivity was normalized to total CaMKIIβ or CaMKIIα levels, respectively. All values were normalized to the mean at PND21, and then expressed as the mean±S.E.M from analyses of the indicated number of animals. * P<0.05, **P<0.01, ***P<0.001.

Spinophilin expression in rodents peaks around PND21 and decreases during aging [34], [41]. Global knockout of spinophilin increases the density of dendritic spines on striatal medium spiny neurons at PND15, but not in adulthood, suggesting that spinophilin plays a key role in developmental “pruning” of spines [42]. In order to begin to understand possible effects of maturation on the spinophilin interactome, we compared striatal Triton-soluble fractions (TSFs) and spinophilin immune complexes from PND21 and adult (PND90-195) mice. Total levels of spinophilin, CaMKIIβ, and PP1 catalytic subunit (PP1c) were significantly reduced in adult TSFs compared to PND21 TSFs, but there was no difference in total levels of CaMKIIα or PSD-95 (Fig. 7A). Reduced levels of both spinophilin and PP1c were immunoprecipitated from adult compared to PND21 striatal TSFs, such that the ratio of PP1 to spinophilin in the complex was not different (Fig. 7B). However, significantly more CaMKIIα and CaMKIIβ co-precipitated with spinophilin from adult compared to PND21 TSFs (Fig. 7B), suggesting that maturation enhances interactions of CaMKII with spinophilin. This enhanced interaction does not appear to be due to increased Thr286 autophosphorylation during maturation (Fig. 7C).

### Maturation increases CaMKII binding to the spinophilin C-terminal domain

**Figure 8:**
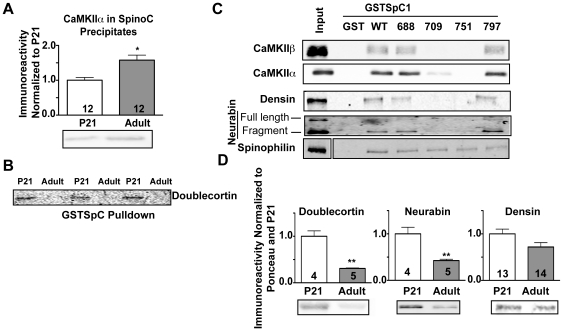
Age-dependent increases in CaMKII binding to the C-terminal domain of spinophilin. *A.* Striatal TSFs isolated from PND21 and adult mice were incubated with GSTSpC. Complexes were isolated using glutathione agarose and immunoblotted for CaMKIIα. *B.* Striatal TSFs isolated from PND21 (P21) and adult animals were incubated with GSTSpC. Complexes were immunoblotted for doublecortin. An N of 3 animals is shown. *C.* Striatal TSFs were incubated with WT or mutated (L688P, L709P, L751P, or L797P) GSTSpC1. Complexes were isolated using glutathione agarose and immunoblotted as indicated. The figure is representative of 2 experiments. *D.* Striatal TSFs isolated from PND21 (P21) and adult animals were immunoblotted for doublecortin, neurabin, or densin. All values were normalized to the mean at PND21, and then expressed as the mean±S.E.M from the indicated number of animals. * P<0.05, **P<0.01.

As an initial probe of the mechanisms underlying increased CaMKII binding to spinophilin during maturation, we compared the binding of proteins in PND21 and adult striatal TSFs to GSTSpC. Notably more CaMKII was precipitated by GSTSpC from adult vs. PND21 fractions suggesting that maturation increases CaMKII interaction with the spinophilin C-terminal domain (Fig. 8A). In contrast, binding of another C-terminal domain SpAP, doublecortin, was decreased in adulthood (Fig. 8B). In order to compare mechanisms underlying association of several striatal SpAPs in the TSF fraction with the C-terminal domain, we examined the effects of mutations that selectively disrupted individual coiled-coil domains in GSTSpC1 (see above). Notably, the L709P and L751P mutations substantially attenuated binding of CaMKII, densin, and neurabin, whereas the L688P and L797P mutations had a reduced effect (Fig. 8C). In contrast, striatal spinophilin bound to all four mutated proteins (Fig. 8C). These data suggest that interactions of several SpAPs with the spinophilin C-terminal domain require intact coiled-coil structures. Interestingly, total levels of several C-terminal domain SpAPs including doublecortin (P<0.01), neurabin (P<0.01), and densin (p = 0.05) were reduced in adult compared to PND21 TSFs (Fig. 8D). Thus, enhanced CaMKII binding to the C-terminal coiled-coil domains of spinophilin with maturation occurs concurrently with reduced expression of other proteins that may compete for the interaction.

### CaMKII binding to the spinophilin N-terminal domain - a role for α-internexin during maturation

**Figure 9:**
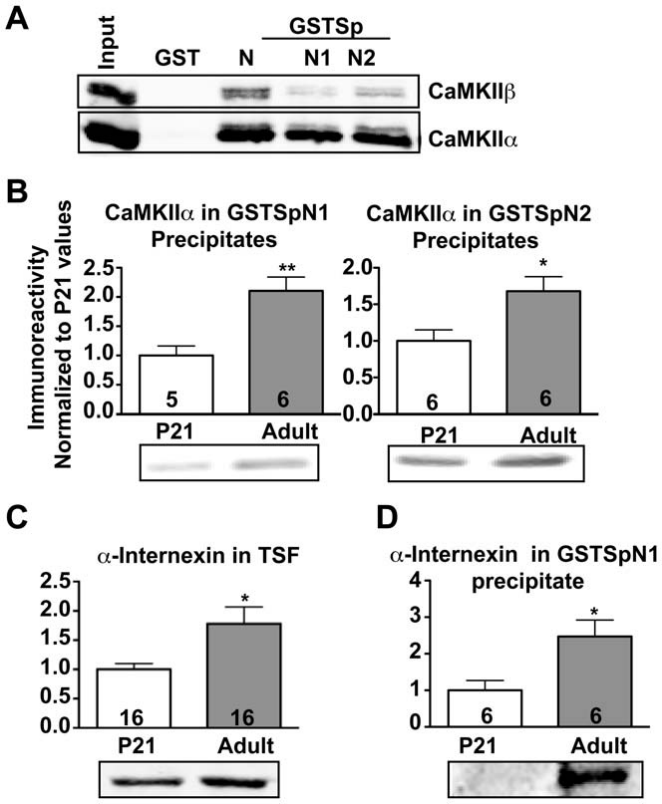
Age-dependent increases in CaMKII binding to the N-terminal domain of spinophilin. *A.* Whole forebrain TSFs were incubated with GST, GSTSpN, GSTSpN1, or GSTSpN2. Complexes were isolated and immunoblotted for both CaMKIIα and CaMKIIβ. *B.* Striatal TSFs isolated from PND21 (P21) and adult animals were incubated with GSTSpN1 or GSTSpN2. Complexes were isolated using glutathione agarose and immunoblotted for CaMKIIα. *C.* Immunoblot of α-internexin from striatal TSFs isolated from PND21 and adult animals. *D.* Striatal TSFs isolated from PND21 (P21) and adult animals were incubated with GSTSpN1. Complexes were isolated and immunoblotted for myosin Va or α-internexin. All values were normalized to the mean at PND21, and then expressed as the mean±S.E.M from the indicated number of animals. * P<0.05, **P<0.01.

Although purified CaMKII binds directly to GSTSpN2, but not to GSPSpN1 (Fig. 1C), we found that GSTSpN1 and GSTSpN2 bound comparable amounts of CaMKII in striatal TSFs (Fig. 9A). These data suggest that other striatal protein(s) may facilitate indirect CaMKII interactions with residues 1–154 of spinophilin. Notably, maturation increased the amount of striatal CaMKII that can associate with both GSTSpN1 and GSTSpN2 (Fig. 9B). Interestingly, total levels of α-internexin, another N-terminal binding SpAP [31] and known CaMKII substrate [43], were increased in adult compared to PND21 striatal TSFs (Fig. 9C). Moreover, the association of striatal α-internexin with GSTSpN1 was significantly increased in adulthood compared to PND21 (Fig. 9D). Thus, increasing levels of α-internexin during maturation may bridge indirect interactions between spinophilin N-terminal domains and CaMKII.

### Age-dependent changes in CaMKII and spinophilin interactomes

**Figure 10:**
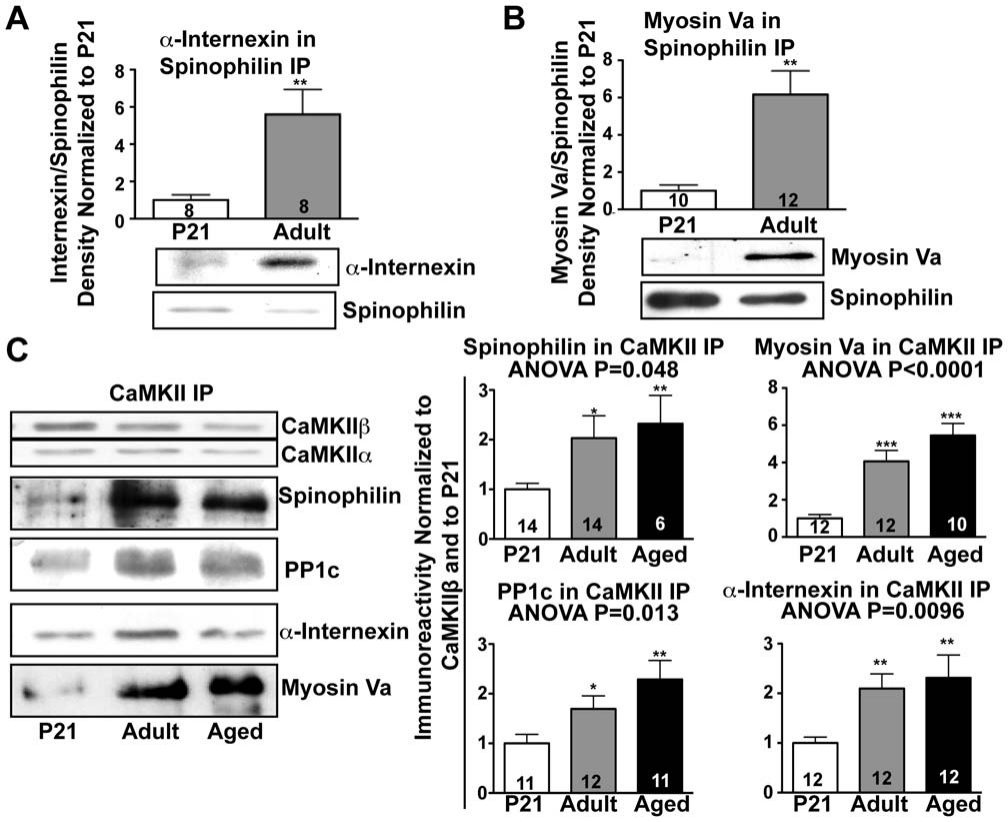
Aging increases the association of spinophilin, PP1, myosin Va, and α-internexin with CaMKII. *A/B.* Spinophilin was immunoprecipitated from striatal TSFs of PND21 or adult mice. Precipitates were immunoblotted for α-internexin (A) or myosin Va (B). *C.* CaMKII was immunoprecipitated from striatal TSFs isolated from PND21 (P21), adult, or aged animals. Precipitates were immunoblotted for CaMKIIβ, CaMKIIα, spinophilin, PP1c, myosin Va, and α-internexin. All values were normalized to the mean at PND21, and then expressed as the mean±S.E.M from the indicated number of animals. * P<0.05, **P<0.01, ***P<0.001.

We investigated whether maturation affects the association of α-internexin with spinophilin in the striatum. Spinophilin immune complexes isolated from adult striatal TSFs contained more α-internexin compared to PND21 immune complexes (Fig. 10A), paralleling the increased association of CaMKII (Fig. 7B). Moreover, adult spinophilin immune complexes contained higher levels of Myosin Va (Fig. 10B), a motor protein that is known to interact with α-internexin [44], spinophilin [31], and CaMKII [45], even though there was no difference in total myosin Va levels between PND21 and adult striatal TSFs (data not shown). Additional co-immunoprecipitation studies showed that CaMKII immune complexes isolated from adult and aged TSFs contained significantly higher levels of spinophilin, PP1c, myosin-Va, and α-internexin than CaMKII complexes isolated from PND21 TSFs (Fig. 10C). Taken together, these data show that normal maturation and aging significantly alters the composition of dendritic signaling protein complexes.

## Discussion

It is well established that spinophilin can act as a scaffold to target PP1 to its substrates [26]. Emerging evidence suggests that CaMKII may also act as a scaffold to target proteins and structures to the PSD [10]. Our present studies suggest an age-dependent mechanism to integrate the actions of these two signaling molecules on common downstream targets associated with their respective complexes.

### Mechanisms of CaMKII-spinophilin interaction

CaMKII binds to PSD proteins by both autophosphorylation-dependent and independent mechanisms [24]. We report here that activated, Thr286-autophosphorylated, but not inactive, CaMKII directly binds to a domain within residues 151–300 of spinophilin in close proximity to the high affinity F-actin binding domain within residues 1–154. F-actin has little effect on direct CaMKII binding to GSTSpN2 (Fig. 1E), but displaces CaMKII from a longer N-terminal domain fragment containing both domains (Fig. 1F). Although these data suggest that the CaMKII interaction with N-terminal domains in spinophilin can be modulated by changes in actin polymerization and the binding of F-actin, we found that the C-terminal domain of spinophilin also directly interact with non-phosphorylated (inactive) CaMKII (Fig. 1C). Coiled-coil motifs in the C-terminal domain are thought to mediate the assembly of dimeric or trimeric forms of spinophilin [34], [46]. Point mutations in GSTSpC1 that are predicted to disrupt individual coiled-coil motifs similarly disrupted the binding of either CaMKII or His-spinophilin. Discrepancies in the relative effects of mutating some of the coiled-coil motifs on interactions with CaMKII or spinophilin when presented as purified proteins (Fig. 3B) or in striatal TSFs (Fig. 8C) presumably reflect the impact of other striatal proteins on these interactions. In combination, these data suggest an important role for the coiled-coil motifs in both oligomerization of spinophilin and in CaMKII binding, but these interactions do not appear to be competitive (Fig. 3D).

Interactions of CaMKII with the NMDAR GluN2B subunit and several other synaptic proteins are dependent in part on CaMKII activation by Ca^2+^/calmodulin-binding and/or Thr286 autophosphorylation [47], [48], [49]. Similarly, we found that CaMKIIα/β activation by Thr286/7 autophosphorylation is required for direct interactions with the N-terminal domain in spinophilin and enhances CaMKII interactions with the C-terminal domain. However, based on prior studies, CaMKII activation by Ca^2+^/calmodulin binding (without autophosphorylation) is also likely to enhance interactions with spinophilin. Nevertheless, spinophilin immune complexes from T286A-KI mice, as well as complexes isolated using GSTSpN (but not GSTSpC) contained less CaMKII than those from WT mice (Fig. 2A), without changes in CaMKIIβ phosphorylation at Thr287 [14], suggesting that Thr286 autophosphorylation modulates CaMKII binding to spinophilin *in vivo*.

### Spinophilin-dependent targeting of PP1 to CaMKII on the F-actin cytoskeleton

A central domain of spinophilin directly binds PP1, targeting it to the actin cytoskeleton in intact cells [37]. Therefore it seems unlikely that PP1 binding to spinophilin will interfere with CaMKII binding to the N- or C-terminal domains. Indeed, the co-expression of spinophilin results in significant co-localization of CaMKII and PP1 in HEK cells (Fig. 4B). Additionally PP1, spinophilin, and CaMKII co-localize in dendritic spines of cultured neurons (Fig. 6 and [19]). Moreover, the association of PP1 and spinophilin with CaMKII in the striatum is strongly enhanced in parallel during maturation and aging. Taken together, our data suggest that spinophilin targets PP1 to CaMKII in an age-dependent manner.

Our data also show that spinophilin can play a role in targeting the two prominent neuronal CaMKII isoforms (CaMKIIα and CaMKIIβ) to F-actin. While previous studies have shown that proteins translated from certain mRNA splice variants of CaMKIIβ expressed in the adult brain directly bind F-actin [50], CaMKIIα, and embryonic CaMKIIβ splice variants, are unable to directly bind F-actin [38], [51]. However, dodecameric CaMKII holoenzymes contain variable mixtures of the isoforms [52], and the relative isoform ratio in each holoenzyme depends on their expression levels, which vary between different neuronal cell types and across development. In adult rodent forebrain, there is approximately 3-fold more CaMKIIα compared to CaMKIIβ [53]. Moreover, CaMKIIα, but not CaMKIIβ, can be locally synthesized in neuronal dendrites [54]. Thus, while some CaMKIIβ variants can target mixed CaMKIIα/β holoenzymes to F-actin, spinophilin appears to play a role in targeting non F-actin binding CaMKII dodecamers to F-actin. For example, the localization of a GFP-tagged CaMKIIβ lacking the F-actin-binding domain to the phalloidin-stained cytoskeleton was significantly enhanced by co-expression of spinophilin (Fig. 5B, C). Since F-actin displaces CaMKII from the N-terminal domain (Fig. 1E), spinophilin-dependent targeting of CaMKII to F-actin is probably due to the association of CaMKII with the C-terminal domain of spinophilin.

F-actin is highly abundant in dendritic spines where it plays a major role modulating synaptic morphology and function. Postsynaptic co-localization of spinophilin with CaMKII is consistent with a role for this interaction in synaptic targeting of CaMKII. However, many other proteins have also been implicated in postsynaptic targeting of CaMKII, including NMDA receptor subunits, densin, and α-actinin [49]. In combination, these data suggest that dendritic spines contain a mixture of CaMKII holoenzyme subpopulations that are associated with different protein complexes. These complexes presumably confer unique regulatory properties on CaMKII or direct CaMKII actions toward distinct downstream targets that are important for different aspects of synaptic regulation.

### Age-dependent changes in the spinophilin interactome

Previous studies using yeast two hybrid screens, cultured cells/neurons, or co-immunoprecipitations from embryonic tissue have identified and validated multiple SpAPs [55]. However, only a subset of the known SpAPs were detected in our previous proteomics screen of spinophilin complexes isolated from adult striatum [31]. We hypothesized that age-dependent and/or tissue-specific differences in the spinophilin interactome accounted for these discrepancies. Indeed the present studies show that association of CaMKII with spinophilin is strongly enhanced during maturation and aging.

Changes in Thr286 autophosphorylation of CaMKIIα do not appear to explain the increased interactions of CaMKII with spinophilin during maturation and aging (Fig. 7C). Rather, multiple mechanisms may be involved; first, interactions of CaMKII with the C-terminal domain of spinophilin increase, perhaps due to reduced expression and decreased competition of SpAPs that also bind to the C-terminal domain by similar mechanisms. Second, CaMKII binding to the N-terminal domain of spinophilin also increases, apparently via a more complex mechanism(s). Our data suggest that other striatal proteins may bridge increased interactions of CaMKII with the N-terminal domains of spinophilin. Candidates for these bridging proteins include several recently identified SpAPs that are also known to associate with CaMKII, including α-actinin, densin, α-internexin, and myosin Va [45], [49], [56]. Consistent with this hypothesis, we found that maturation increased the association of α-internexin and myosin Va with spinophilin immune complexes (Fig. 10A, 10B), and that maturation and aging also increased the levels of spinophilin, PP1, myosin Va, and α-internexin in CaMKII immune complexes. Taken together, our data suggest that maturation/aging enhances the spinophilin-dependent targeting of both CaMKII and PP1 to F-actin and other associated proteins.

### Functional implications of the spinophilin/CaMKII interaction

Balanced CaMKII and PP1 activities maintain normal phosphorylation/dephosphorylation of several PSD proteins that play critical roles in modulating dendritic spine number/morphology and function [57]. While it is well established that A-kinase anchoring proteins coordinate the activities of protein kinase A with opposing phosphatases [58], the present data are the first to suggest the coordination of CaMKII activity with an opposing protein phosphatase (PP1) via a scaffolding protein (spinophilin). Among dendritic substrates that may be targeted by this complex is CaMKII itself. CaMKII dephosphorylation can be modulated by PSD targeting, and PSD-associated CaMKII appears to be preferentially dephosphorylated by PP1 [13], [59]. While this may initially seem at odds with *in vitro* observations that spinophilin inhibits PP1 [60], recent studies suggest that the inhibition is substrate selective [61]. Since Thr286 autophosphorylation is largely unaltered by maturation and aging despite the robust increases in levels of the CaMKII-spinophilin complex, we speculate that the PP1 activity toward phospho-Thr286 in CaMKII is inhibited in the spinophilin complex and that PP1 may dephosphorylate other sites in CaMKII. This model appears consistent with a recent study showing that phospho-Thr286 in PSD-associated CaMKII is protected from PP1 dephosphorylation [62]. Future studies need to determine the role of spinophilin in modulating PP1-dependent dephosphorylation of specific sites on CaMKII during maturation.

Alternatively, CaMKII binding to spinophilin may enhance phosphorylation of the N-terminal domain of spinophilin itself to decrease interactions with F-actin [63], [64]. Also, the binding to spinophilin may target CaMKII to phosphorylate additional SpAPs that can modulate the F-actin cytoskeleton such as Tiam1 or Kalirin-7 [55], [65], [66]. Notably, CaMKII phosphorylation of Kalirin-7 is required for activity-dependent AMPA receptor insertion and associated changes in dendritic spine morphology [66].

### The spinophilin/CaMKII interactome in disease

Striatal dopamine depletion in animal models of PD increases CaMKIIα autophosphorylation at Thr286 [23], [41] in parallel with decreased PP1γ1 activity and increased PP1γ1 binding to spinophilin [22]. Interestingly, phosphorylation of Ser831 in AMPAR GluA1 subunits, a downstream target of both CaMKII and PP1, is only increased following prolonged (>9 months) dopamine depletion [41]. Since total levels of spinophilin are reduced during aging [34], [41], CaMKII-spinophilin complexes described herein may modulate the downstream consequences of long-term dopamine depletion. Future studies will need to define the role of spinophilin in modulating the phosphorylation of Ser831 in GluA1.

Recent studies of a mouse model of typical α-thalassemia X-linked mental retardation (ATRX) also demonstrated increased autophosphorylation of CaMKIIα at Thr286, decreased levels of spinophilin and PP1, and increased phosphorylation of two downstream CaMKII/PP1 substrates, Tiam1 and Kalirin-7 [67]. The pathological convergence of these two signaling pathways in adult ATRX mice correlated with altered spine morphology, suggesting a role for this complex in regulating adult dendritic spine morphology.

### Final summary

Here we report that spinophilin can directly bind CaMKII, suggesting a mechanism for the formation of a “scaffold of scaffolds”. This multi-protein complex may couple CaMKII and PP1 within defined subcellular microdomains, providing precise control of the phosphorylation/dephosphorylation of common, co-localized substrates

## Materials and Methods

### Animals

This study was carried out in strict accordance with the recommendations in the Guide for the Care and Use of Laboratory Animals of the National Institutes of Health. The protocol was approved by the Vanderbilt Institutional Animal Care and Use Committee (Protocol #'s M/08/101 and M/05/349). Male or female PND20-PND21, male 2.75–9 month old (adult), or male 14.5–24 month old (aged) mice were sacrificed and neostriatum (referred to as striatum) was dissected and either used fresh or frozen on dry ice.

### Antibodies

The goat CaMKII antibody was previously described [68]. A list of the commercially available antibodies used is given in Table S1.

### GST proteins

GST spinophilin fusion protein containing residues 1–300, 446–691 or 446–817 were made as described previously [31], [46], [69]. Additional fusion proteins were created in pGEX4T-1. Point mutations in GST-spinophilin proteins were created using QuickChange site-directed mutagenesis (Stratagene, Santa Clara, CA). Full-length His-tagged spinophilin construct was inserted into the pRSET A vector (Invitrogen, Grand Island, NY). Primer sequences and restriction sites used for novel constructs are listed in Table S2.

### Mammalian expression constructs

Generation of myc-tagged spinophilin has been previously described [46]. WT and ΔABD (355–392 deletion mutant), GFP-tagged rat CaMKIIβ were kind gifts of Dr. L. Redmond Hardy (Medical College of Georgia). Untagged CaMKIIβ was created by PCR amplification of the full length GFP-tagged CaMKIIβ and shuttling the PCR product into the pCDNA3.1+ vector (Invitrogen) between EcoRI and XhoI sites. Untagged mouse CaMKIIα in pcDNA3.1+ has been previously described [70].

### Autophosphorylation of CaMKII isoforms

Murine CaMKIIα and Xenopus CaMKIIβ, were purified from baculovirus-infected Sf9 cells and selectively autophosphorylated at Thr286/7 as described [68], [71].

### Actin polymerization

Rabbit skeletal muscle actin (250 µg; Catalog# AKL-99A; Cytoskeleton Inc, Denver, CO) was resuspended in 25 µl of water, and then mixed on ice for 1 h with 600 µl of Buffer A (5 mM Tris-HCl pH 8.0, 0.2 mM CaCl_2_, 0.2 mM ATP). F-actin was polymerized by adding KCl, MgCl2, and ATP to final concentrations of 50, 2, and 1 mM, respectively and incubated for an additional 1 h at room temperature while mixing by rotation. The polymerized F-actin was stored at 4°C and used within one month.

### Tissue Homogenization

Two frozen mouse striata (one from each hemisphere; ∼20 mg total tissue) or 1/2 forebrain (∼200 mg) were homogenized in 2 ml of a low ionic strength (2 mM Tris-HCl pH 7.4, 2 mM EDTA, 2 mM EGTA, 1 mM DTT, 0.2 mM PMSF, 1 mM benzamidine, 10 µg/ml leupeptin, 40 µg/ml Soybean Trypsin Inhibitor, 10 µM pepstatin, and 1 µM microcystin) buffer with 1% Triton X-100 using a Teflon-glass Wheaton tissue grinder (Wheaton Science products, Millville, NJ) with motorized plunger and incubated at 4°C for 30–60 min. Samples were adjusted to 0.8–1.2 mg/ml total protein and then centrifuged at 9,000× g at 4°C for 10 min. The TSF supernatant was used for immunoprecipitation as described previously [22].

### Immunoprecipitations

Immunoprecipitates from striatal TSFs were performed as previously described [22] using 3.75 µg of the mouse CaMKIIβ antibody or 4 µg of the goat spinophilin antibody.

### GST co-sedimentation

GST constructs (10 µg for striatal lysates, 250 nM for *in vitro* studies) were incubated with either 350–500 µl of striatal TSFs, autophosphorylated or non-autophosphorylated, purified CaMKII (see above), full-length His-tagged spinophilin, or F-actin in 300–500 µl of GST-Pulldown buffer (50 mM Tris-HCl pH 7.5, 200 mM NaCl, 0.5% Triton X-100) for 60 min. 40 µl of a 1∶1 slurry of glutathione agarose beads (Sigma) were added and incubated for 2 hr - overnight (*in vitro*) or overnight (striatal lysates). Samples were washed and prepared as previously described [31].

### Semi-quantitative immunoblot analysis

Immunoblot analysis was done similar to as described [22]. Nitrocellulose membranes were blocked in either 5% milk in TBST or StartingBlock blocking buffer (Thermo-Fisher) and incubated with the appropriate primary and secondary antibodies. For detection with X-ray film, HRP-conjugated secondary antibodies (SantaCruz) were developed with Western Lightning chemiluminescent reagent (PerkinElmer, Waltham, MA). For detection using the Odyssey system (LiCor Biosciences, Lincoln, NE), infrared-conjugated secondary antibodies (LiCor) were used. Densitometry was performed using Image J (National Institutes of Health, Bethesda, MD) on images linearly adjusted for brightness and contrast. For quantitation of TSFs, signals were normalized for protein loading by dividing the individual band area by Ponceau S staining. For co-immunoprecipitation studies, individual co-precipitating proteins were normalized to the amount of immunoprecipitated protein (e.g. CaMKII, spinophilin). To normalize across gels, a ratio was calculated by dividing each value from the average of the PND21 group on the corresponding gel.

### Co-localization in cells

WT, untagged CaMKIIβ, GFP-tagged CaMKIIβ ΔABD, myc-spinophilin, and/or GFP-PP1γ1 were transfected into HEK293 cells using Lipofectamine LTX (Invitrogen) or PolyJet (SignaGen, Rockville, MD) transfection reagents as previously described [31]. Cells were either incubated with PBS containing EGTA for 5 minutes or directly removed for immunocytochemistry and intensity correlation quotient (ICQ) analysis as previously described [31], [72]. Alexa Fluor 633-conjugated phalloidin (Invitrogen) was used to stain all cells for endogenous F-actin.

### Immunohistochemistry of cortical cultures

Rat cortical cultures were prepared, stained, and imaged after 15 days *in vitro* as previously described [19].

### Statistical analyses

All statistical analyses were performed in Prism (GraphPad, La Jolla, CA). A student's t-test was used to compare two groups. For comparisons of three groups, an ANOVA was performed followed by a post-hoc student's t-test if the ANOVA was significant (P<0.05).

## Supporting Information

Figure S1 **Disruption of Spinophilin Coiled-Coil Structure by Leucine to Proline Mutations.** *A.* Predicted selective disruption of individual coiled-coil domains by leucine to proline mutations. All structural predictions were performed using Multicoil. A. L688P. *B.* L709P. *C.* L751P. *D.* L798P.
(TIF)

Figure S2 **Additional confocal images (compressed z-stacks) of HEK cells expressing CaMKIIβ and PP1γ1.**
(TIF)

Figure S3 **Additional confocal images (compressed z-stacks) of HEK cells expressing CaMKIIβ, PP1γ1, and myc-spinophilin.**
(TIF)

Table S1 **List of antibodies, vendors, and catalog numbers.**
(XLSX)

Table S2 **Primer list for GST spinophilin constructs.**
(XLSX)

## References

[1] Zhou Q, Homma KJ, Poo MM (2004). Shrinkage of dendritic spines associated with long-term depression of hippocampal synapses.. Neuron.

[2] Levine MS, Adinolfi AM, Fisher RS, Hull CD, Buchwald NA (1986). Quantitative morphology of medium-sized caudate spiny neurons in aged cats.. Neurobiol Aging.

[3] Levine MS, Fisher RS, Hull CD, Buchwald NA (1986). Postnatal development of identified medium-sized caudate spiny neurons in the cat.. Brain Res.

[4] Tada T, Sheng M (2006). Molecular mechanisms of dendritic spine morphogenesis.. Curr Opin Neurobiol.

[5] Holtmaat A, Svoboda K (2009). Experience-dependent structural synaptic plasticity in the mammalian brain.. Nat Rev Neurosci.

[6] Sheng M, Hoogenraad CC (2007). The postsynaptic architecture of excitatory synapses: a more quantitative view.. Annu Rev Biochem.

[7] Cheng D, Hoogenraad CC, Rush J, Ramm E, Schlager MA (2006). Relative and absolute quantification of postsynaptic density proteome isolated from rat forebrain and cerebellum.. Mol Cell Proteomics.

[8] Barria A, Muller D, Derkach V, Griffith LC, Soderling TR (1997). Regulatory phosphorylation of AMPA-type glutamate receptors by CaM-KII during long-term potentiation.. Science.

[9] Fukunaga K, Stoppini L, Miyamoto E, Muller D (1993). Long-term potentiation is associated with an increased activity of Ca2+/calmodulin-dependent protein kinase II.. J Biol Chem.

[10] Bingol B, Wang CF, Arnott D, Cheng D, Peng J (2010). Autophosphorylated CaMKIIalpha acts as a scaffold to recruit proteasomes to dendritic spines.. Cell.

[11] Giese KP, Fedorov NB, Filipkowski RK, Silva AJ (1998). Autophosphorylation at Thr286 of the alpha calcium-calmodulin kinase II in LTP and learning.. Science.

[12] Hudmon A, Schulman H (2002). Neuronal CA2+/calmodulin-dependent protein kinase II: the role of structure and autoregulation in cellular function.. Annu Rev Biochem.

[13] Strack S, Choi S, Lovinger DM, Colbran RJ (1997). Translocation of autophosphorylated calcium/calmodulin-dependent protein kinase II to the postsynaptic density.. J Biol Chem.

[14] Gustin RM, Shonesy BC, Robinson SL, Rentz TJ, Baucum AJ (2011). Loss of Thr286 phosphorylation disrupts synaptic CaMKIIalpha targeting, NMDAR activity and behavior in pre-adolescent mice.. Mol Cell Neurosci.

[15] Zhang GR, Cheng XR, Zhou WX, Zhang YX (2009). Age-related expression of calcium/calmodulin-dependent protein kinase II A in the hippocampus and cerebral cortex of senescence accelerated mouse prone/8 mice is modulated by anti-Alzheimer's disease drugs.. Neuroscience.

[16] Brocke L, Srinivasan M, Schulman H (1995). Developmental and regional expression of multifunctional Ca2+/calmodulin-dependent protein kinase isoforms in rat brain.. J Neurosci.

[17] Bodhinathan K, Kumar A, Foster TC (2010). Intracellular redox state alters NMDA receptor response during aging through Ca2+/calmodulin-dependent protein kinase II.. J Neurosci.

[18] Ouimet CC, da Cruz e Silva EF, Greengard P (1995). The alpha and gamma 1 isoforms of protein phosphatase 1 are highly and specifically concentrated in dendritic spines.. Proc Natl Acad Sci U S A.

[19] Strack S, Kini S, Ebner FF, Wadzinski BE, Colbran RJ (1999). Differential cellular and subcellular localization of protein phosphatase 1 isoforms in brain.. J Comp Neurol.

[20] Blitzer RD, Connor JH, Brown GP, Wong T, Shenolikar S (1998). Gating of CaMKII by cAMP-regulated protein phosphatase activity during LTP.. Science.

[21] Norris CM, Halpain S, Foster TC (1998). Alterations in the balance of protein kinase/phosphatase activities parallel reduced synaptic strength during aging.. J Neurophysiol.

[22] Brown AM, Baucum AJ, Bass MA, Colbran RJ (2008). Association of protein phosphatase 1 gamma 1 with spinophilin suppresses phosphatase activity in a Parkinson disease model.. J Biol Chem.

[23] Picconi B, Gardoni F, Centonze D, Mauceri D, Cenci MA (2004). Abnormal Ca2+-calmodulin-dependent protein kinase II function mediates synaptic and motor deficits in experimental parkinsonism.. J Neurosci.

[24] Colbran RJ (2004). Targeting of calcium/calmodulin-dependent protein kinase II.. Biochem J.

[25] Bollen M, Peti W, Ragusa MJ, Beullens M (2010). The extended PP1 toolkit: designed to create specificity.. Trends Biochem Sci.

[26] Terry-Lorenzo RT, Elliot E, Weiser DC, Prickett TD, Brautigan DL (2002). Neurabins recruit protein phosphatase-1 and inhibitor-2 to the actin cytoskeleton.. J Biol Chem.

[27] Buchsbaum RJ, Connolly BA, Feig LA (2003). Regulation of p70 S6 kinase by complex formation between the Rac guanine nucleotide exchange factor (Rac-GEF) Tiam1 and the scaffold spinophilin.. J Biol Chem.

[28] Ryan XP, Alldritt J, Svenningsson P, Allen PB, Wu GY (2005). The Rho-specific GEF Lfc interacts with neurabin and spinophilin to regulate dendritic spine morphology.. Neuron.

[29] Tolias KF, Bikoff JB, Kane CG, Tolias CS, Hu L (2007). The Rac1 guanine nucleotide exchange factor Tiam1 mediates EphB receptor-dependent dendritic spine development.. Proc Natl Acad Sci U S A.

[30] Penzes P, Johnson RC, Sattler R, Zhang X, Huganir RL (2001). The neuronal Rho-GEF Kalirin-7 interacts with PDZ domain-containing proteins and regulates dendritic morphogenesis.. Neuron.

[31] Baucum AJ, Jalan-Sakrikar N, Jiao Y, Gustin RM, Carmody LC (2010). Identification and validation of novel spinophilin-associated proteins in rodent striatum using an enhanced ex vivo shotgun proteomics approach.. Mol Cell Proteomics.

[32] Satoh A, Nakanishi H, Obaishi H, Wada M, Takahashi K (1998). Neurabin-II/spinophilin. An actin filament-binding protein with one pdz domain localized at cadherin-based cell-cell adhesion sites.. J Biol Chem.

[33] Barnes AP, Smith FD, VanDongen HM, VanDongen AM, Milgram SL (2004). The identification of a second actin-binding region in spinophilin/neurabin II.. Brain Res Mol Brain Res.

[34] Allen PB, Ouimet CC, Greengard P (1997). Spinophilin, a novel protein phosphatase 1 binding protein localized to dendritic spines.. Proc Natl Acad Sci U S A.

[35] Cheng HY, Schiavone AP, Smithgall TE (2001). A point mutation in the N-terminal coiled-coil domain releases c-Fes tyrosine kinase activity and survival signaling in myeloid leukemia cells.. Mol Cell Biol.

[36] Wolf E, Kim PS, Berger B (1997). MultiCoil: a program for predicting two- and three-stranded coiled coils.. Protein Sci.

[37] Carmody LC, Baucum AJ, Bass MA, Colbran RJ (2008). Selective targeting of the gamma1 isoform of protein phosphatase 1 to F-actin in intact cells requires multiple domains in spinophilin and neurabin.. Faseb J.

[38] Fink CC, Bayer KU, Myers JW, Ferrell JE, Schulman H (2003). Selective regulation of neurite extension and synapse formation by the beta but not the alpha isoform of CaMKII.. Neuron.

[39] Lin YC, Redmond L (2008). CaMKIIbeta binding to stable F-actin in vivo regulates F-actin filament stability.. Proc Natl Acad Sci U S A.

[40] Okamoto K, Narayanan R, Lee SH, Murata K, Hayashi Y (2007). The role of CaMKII as an F-actin-bundling protein crucial for maintenance of dendritic spine structure.. Proc Natl Acad Sci U S A.

[41] Brown AM, Deutch AY, Colbran RJ (2005). Dopamine depletion alters phosphorylation of striatal proteins in a model of Parkinsonism.. Eur J Neurosci.

[42] Feng J, Yan Z, Ferreira A, Tomizawa K, Liauw JA (2000). Spinophilin regulates the formation and function of dendritic spines.. Proc Natl Acad Sci U S A.

[43] Yoshimura Y, Yamauchi T (1997). Phosphorylation-dependent reversible association of Ca2+/calmodulin-dependent protein kinase II with the postsynaptic densities.. J Biol Chem.

[44] Rao MV, Mohan PS, Kumar A, Yuan A, Montagna L (2011). The myosin Va head domain binds to the neurofilament-L rod and modulates endoplasmic reticulum (ER) content and distribution within axons.. PLoS One.

[45] Costa MC, Mani F, Santoro W, Espreafico EM, Larson RE (1999). Brain myosin-V, a calmodulin-carrying myosin, binds to calmodulin-dependent protein kinase II and activates its kinase activity.. J Biol Chem.

[46] MacMillan LB, Bass MA, Cheng N, Howard EF, Tamura M (1999). Brain actin-associated protein phosphatase 1 holoenzymes containing spinophilin, neurabin, and selected catalytic subunit isoforms.. J Biol Chem.

[47] Jiao Y, Jalan-Sakrikar N, Robison AJ, Baucum AJ, Bass MA (2011). Characterization of a central Ca2+/calmodulin-dependent protein kinase IIalpha/beta binding domain in densin that selectively modulates glutamate receptor subunit phosphorylation.. J Biol Chem.

[48] Nikandrova YA, Jiao Y, Baucum AJ, Tavalin SJ, Colbran RJ (2010). Ca2+/calmodulin-dependent protein kinase II binds to and phosphorylates a specific SAP97 splice variant to disrupt association with AKAP79/150 and modulate alpha-amino-3-hydroxy-5-methyl-4-isoxazolepropionic acid-type glutamate receptor (AMPAR) activity.. J Biol Chem.

[49] Robison AJ, Bass MA, Jiao Y, MacMillan LB, Carmody LC (2005). Multivalent interactions of calcium/calmodulin-dependent protein kinase II with the postsynaptic density proteins NR2B, densin-180, and alpha-actinin-2.. J Biol Chem.

[50] O'Leary H, Lasda E, Bayer KU (2006). CaMKIIbeta association with the actin cytoskeleton is regulated by alternative splicing.. Mol Biol Cell.

[51] Sanabria H, Swulius MT, Kolodziej SJ, Liu J, Waxham MN (2009). {beta}CaMKII regulates actin assembly and structure.. J Biol Chem.

[52] Woodgett JR, Davison MT, Cohen P (1983). The calmodulin-dependent glycogen synthase kinase from rabbit skeletal muscle. Purification, subunit structure and substrate specificity.. Eur J Biochem.

[53] Erondu NE, Kennedy MB (1985). Regional distribution of type II Ca2+/calmodulin-dependent protein kinase in rat brain.. J Neurosci.

[54] Benson DL, Gall CM, Isackson PJ (1992). Dendritic localization of type II calcium calmodulin-dependent protein kinase mRNA in normal and reinnervated rat hippocampus.. Neuroscience.

[55] Sarrouilhe D, di Tommaso A, Metaye T, Ladeveze V (2006). Spinophilin: from partners to functions.. Biochimie.

[56] Yoshimura Y, Aoi C, Yamauchi T (2000). Investigation of protein substrates of Ca(2+)/calmodulin-dependent protein kinase II translocated to the postsynaptic density.. Brain Res Mol Brain Res.

[57] Lisman JE, Zhabotinsky AM (2001). A model of synaptic memory: a CaMKII/PP1 switch that potentiates transmission by organizing an AMPA receptor anchoring assembly.. Neuron.

[58] Logue JS, Scott JD (2010). Organizing signal transduction through A-kinase anchoring proteins (AKAPs).. FEBS J.

[59] Dosemeci A, Reese TS (1993). Inhibition of endogenous phosphatase in a postsynaptic density fraction allows extensive phosphorylation of the major postsynaptic density protein.. J Neurochem.

[60] Hsieh-Wilson LC, Allen PB, Watanabe T, Nairn AC, Greengard P (1999). Characterization of the neuronal targeting protein spinophilin and its interactions with protein phosphatase-1.. Biochemistry.

[61] Ragusa MJ, Dancheck B, Critton DA, Nairn AC, Page R (2010). Spinophilin directs protein phosphatase 1 specificity by blocking substrate binding sites.. Nat Struct Mol Biol.

[62] Mullasseril P, Dosemeci A, Lisman JE, Griffith LC (2007). A structural mechanism for maintaining the ‘on-state’ of the CaMKII memory switch in the post-synaptic density.. J Neurochem.

[63] Hsieh-Wilson LC, Benfenati F, Snyder GL, Allen PB, Nairn AC (2003). Phosphorylation of spinophilin modulates its interaction with actin filaments.. J Biol Chem.

[64] Grossman SD, Futter M, Snyder GL, Allen PB, Nairn AC (2004). Spinophilin is phosphorylated by Ca2+/calmodulin-dependent protein kinase II resulting in regulation of its binding to F-actin.. J Neurochem.

[65] Fleming IN, Elliott CM, Buchanan FG, Downes CP, Exton JH (1999). Ca2+/calmodulin-dependent protein kinase II regulates Tiam1 by reversible protein phosphorylation.. J Biol Chem.

[66] Xie Z, Srivastava DP, Photowala H, Kai L, Cahill ME (2007). Kalirin-7 controls activity-dependent structural and functional plasticity of dendritic spines.. Neuron.

[67] Shioda N, Beppu H, Fukuda T, Li E, Kitajima I (2011). Aberrant Calcium/Calmodulin-Dependent Protein Kinase II (CaMKII) Activity Is Associated with Abnormal Dendritic Spine Morphology in the ATRX Mutant Mouse Brain.. J Neurosci.

[68] McNeill RB, Colbran RJ (1995). Interaction of autophosphorylated Ca2+/calmodulin-dependent protein kinase II with neuronal cytoskeletal proteins. Characterization of binding to a 190-kDa postsynaptic density protein.. J Biol Chem.

[69] Carmody LC, Bauman PA, Bass MA, Mavila N, DePaoli-Roach AA (2004). A protein phosphatase-1gamma1 isoform selectivity determinant in dendritic spine-associated neurabin.. J Biol Chem.

[70] Jiao Y, Robison AJ, Bass MA, Colbran RJ (2008). Developmentally regulated alternative splicing of densin modulates protein-protein interaction and subcellular localization.. J Neurochem.

[71] Brickey DA, Colbran RJ, Fong YL, Soderling TR (1990). Expression and characterization of the alpha-subunit of Ca2+/calmodulin-dependent protein kinase II using the baculovirus expression system.. Biochem Biophys Res Commun.

[72] Li Q, Lau A, Morris TJ, Guo L, Fordyce CB (2004). A syntaxin 1, Galpha(o), and N-type calcium channel complex at a presynaptic nerve terminal: analysis by quantitative immunocolocalization.. J Neurosci.

