# Supplementary figures and images for: Age-Dependent Targeting of Protein Phosphatase 1 to Ca2+/Calmodulin-Dependent Protein Kinase II by Spinophilin in Mouse Striatum
Source: PLoS One. 2012 Feb 13;7(2):e31554. doi: 10.1371/journal.pone.0031554 (PMC3278457; doi:10.1371/journal.pone.0031554)

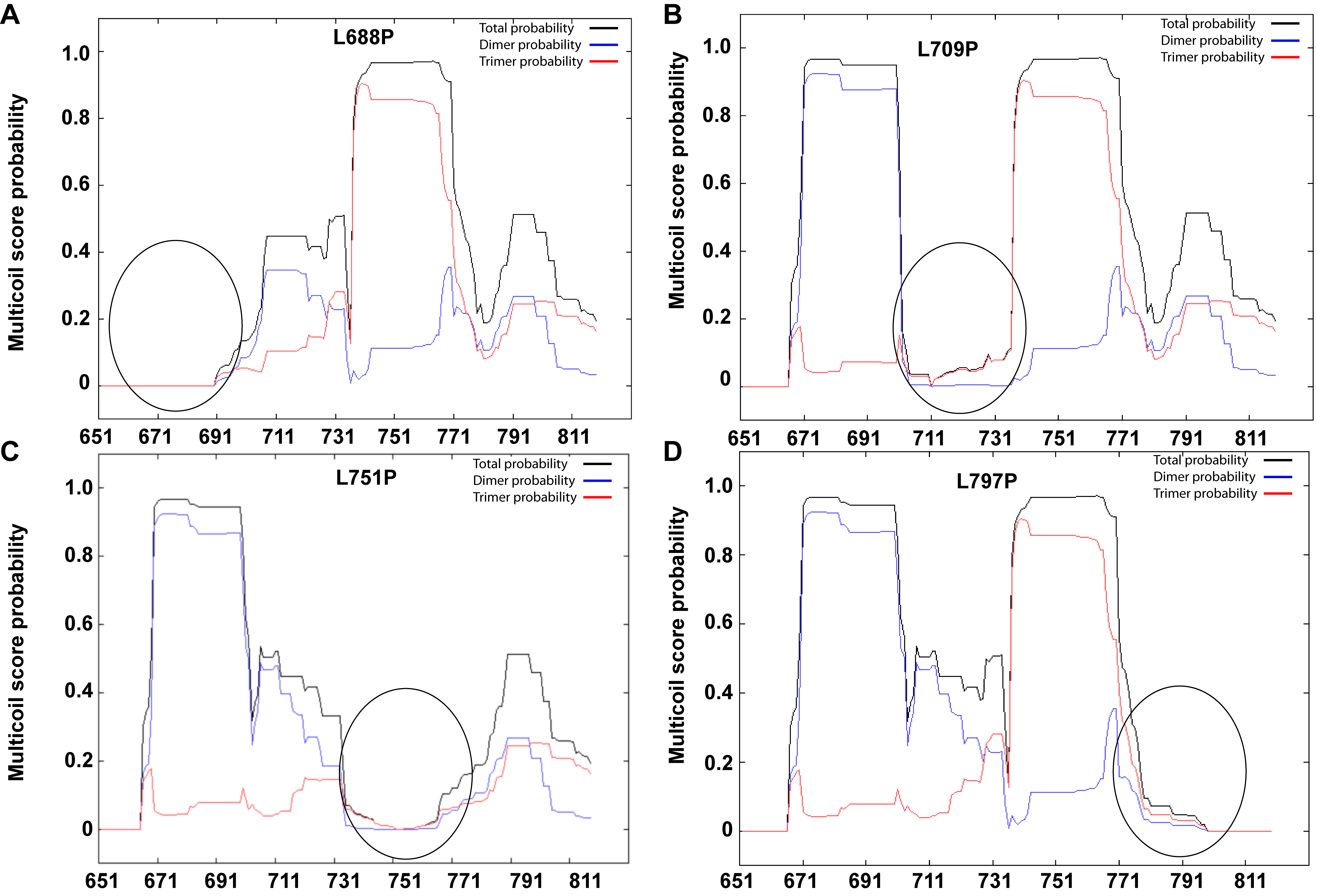

Supplement: Figure S1 — Disruption of Spinophilin Coiled-Coil Structure by Leucine to Proline Mutations. A. Predicted selective disruption of individual coiled-coil domains by leucine to proline mutations. All structural predictions were performed using Multicoil. A. L688P. B. L709P. C. L751P. D. L798P. (TIF) [file pone.0031554.s001.tif]

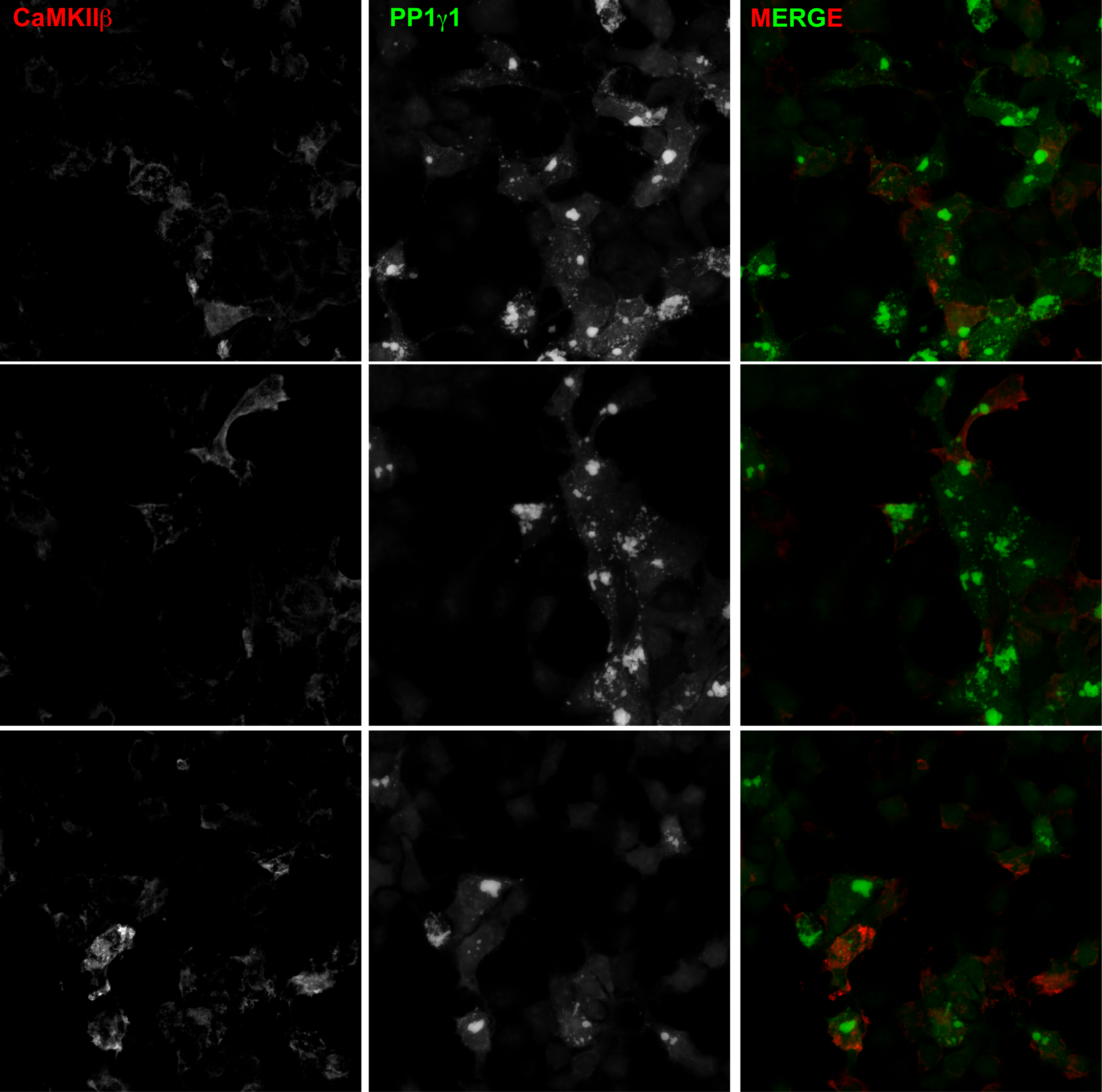

Supplement: Figure S2 — Additional confocal images (compressed z-stacks) of HEK cells expressing CaMKIIβ and PP1γ1. (TIF) [file pone.0031554.s002.tif]

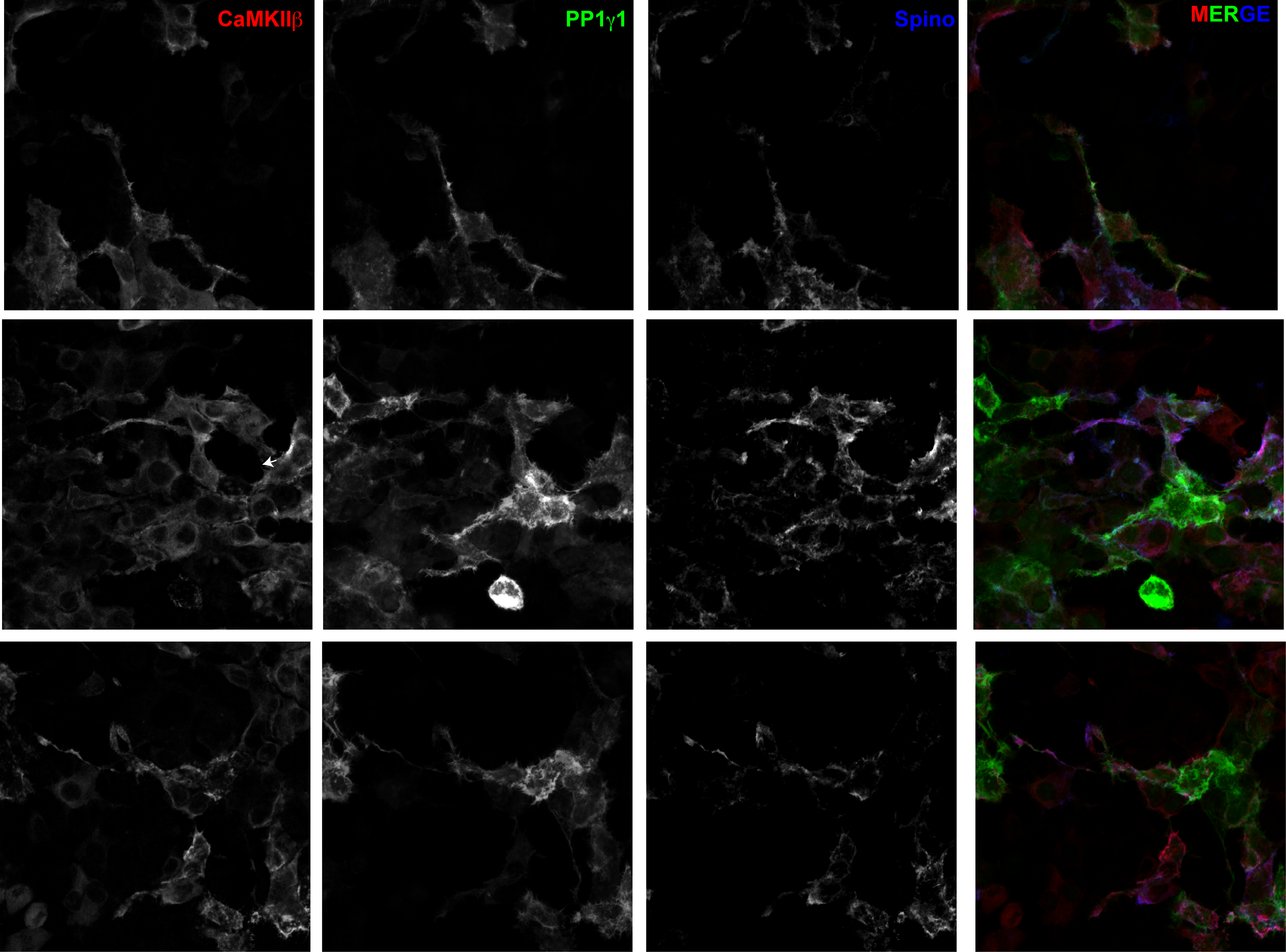

Supplement: Figure S3 — Additional confocal images (compressed z-stacks) of HEK cells expressing CaMKIIβ, PP1γ1, and myc-spinophilin. (TIF) [file pone.0031554.s003.tif]
